# Injection of Air into Abscesses and Fistulæ

**Published:** 1866-02

**Authors:** 


INJECTION OF AIR INTO ABCESSES AND FISTULÆ.—Prof. Rozer states that there is no better means of treating an abscess, the contents of which has become foul and stinking, than by the frequent injection of air by means of an elastic catheter and syringe. The patient is to be instructed to repeat this injection sufficiently often to prevent the accumulation of pus: and this operation not only adds much to comfort by removing the nauseous smells, but also seems to greatly expedite the contraction and closure of the cavity of the abscess. It is of especial benefit in the treatment of the fistulous openings remaining after emphysema, and attended with such a digusting smell. This is speedily removed by aid of the injections, and the healing of the fistula promoted. This is often retarded for months, owing to the valvular condition of the track of the fistula preventing the free issue of the pus, which is secured by the daily passage of the catheter for the purpose of injecting the air.—*Archiv. der Heilkunde*, in *Medical Times and Gazette.*

